# Annotations

**Published:** 1901-05-25

**Authors:** 


### ANNOTATIONS.

### Ice Creams.

The first touch of warm weather brings out the Italian ice-cream vendor, and it becomes a serious question whether more might not be done than can be effected under existing rules and regulations to ensure the purity of the wares he deals in. It is not worth while to repeat what has been said so often as to the offensive condition in which, in too many cases, penny ices are made and stored, but it is important to repeat that there is every reason for believing that the semi-frozen mess which is eaten with such avidity by children in all the poorer quarters of the metropolis is responsible for much disease. It is quite certain that the mere freezing to which these ices are subjected has no effect in hindering their capacity for acting as disease carriers, while no one can watch the conditions under which they are often sold without observing the facilities thereby offered for diffusing infection from mouth to mouth. Eaten in the small quantity in which these ices are generally partaken of we do not think that the coldness of the chilly morsel can often be regarded as the cause of any injury, although we cannot acquit the large portions of ice puddings sometimes taken at the end of a big dinner, even by intelligent people, as being entirely devoid of danger on this score. The risk to the children who on a hot afternoon swarm around the ice-man's barrow arises partly from the microbes, pathogenic and others, derived from the surroundings among which the "cream" has been prepared, and partly from the passage of infection from mouth to mouth owing to the imperfect cleansing to which the glasses are subjected. Certainly the Corporation of Brighton has done well in endeavouring to introduce into a local Act a clause giving them power to regulate the details of the ice-cream trade.

### Training in Mental Diseases.

In addressing a recent meeting of the Medico-Psychological Association on the importance of teaching the subject of insanity to medical students, Dr. Robert Jones had a comparatively easy task; for as it is the fact that it is in the earlier stages of insanity that treatment is most likely to be efficacious (the stages in which the disease comes almost exclusively under the care of the general practitioner) it almost goes without saying that unless the subject is properly taught in the schools, full advantage is never likely to be taken of what is in truth the most important moment in the course of the disease so far as treatment and prevention are concerned. Even were the duty of the practitioner merely confined to the giving of certificates it would be no small responsibility, for as Dr. Jones points out there are nearly 145,000 persons in Great Britain and Ireland who are insane under more than 150,000 certificates, giving an average of five certificates to every member of our profession. If one recognises that on an average, year in year out, each medical man is responsible for a share in keeping five individuals deprived of their liberty, and in throwing the support of all these people upon the rates, it becomes a very serious matter, not merely to doctors but to the public also, that care shall be taken that medical men shall possess some reasonable amount of knowledge concerning this part of their business. Again it is asserted that for every certified insane person there is probably another who is either suffering from a threatened attack, or who, although insane may be harmless, and as to whose treatment his medical attendant is expected to give the best advice. The practitioner is also constantly confronted with grave mental symptoms arising in the course of bodily disease, and these cases require home treatment. Resolve as much as he likes to steer clear of lunacy no medical practitioner can avoid having to do with a good deal of brain disease. But after all one of the chief reasons why the medical student should be thoroughly instructed in this subject is that insanity is best and most successfully treated in its early stages—the stage of abstraction, reverie, papillary changes, insomnia, and capricious appetite. We need not go further. Dr. Robert Jones shows, as indeed he could hardly fail to show, the supreme importance of those who claim to treat disease knowing somewhat about diseases of the mind. If he had chosen to go on to show the perfunctory manner in which this great subject is at present dealt with, and had poured out all the sarcasm of which his tongue was capable upon the lamentable unpreparedness with which the newly fledged practitioner may have to encounter his first mental case and may have to take his share in depriving some unfortunate fellow creature of his liberty, he might perhaps have produced an even more striking presentment of the situation than he has done.

### Plague the Mysterious!

Either there is something wrong about the methods of disinfection commonly adopted for preventing the spread of plague, or else disinfection is much less effectual in controlling an epidemic than most sanitarians would have us believe. Wherein lies the defect we will not presume to say, but there seems no doubt that the efforts made and the money spent last year in attempting to disinfect the slums of Calcutta—efforts which it was hoped would prevent the spread of the disease during the present "plague season" have had but little if any effect upon it. Anyway the plague has this season been worse than ever, and it is stated that cases have cropped up time after time in houses which have been more than once disinfected. To say the least of it, it is a curious and noteworthy fact as showing the limitations of human knowledge, that although we reckon to understand all about the disease itself, and all about the microbe by which it is caused, and although we are able if we so desire to propagate the malady to any extent we like, yet, do as we will, we are not able to lay our fingers in any useful fashion on the track by which the infection gains access to man. That rats have something to do with it seems plain; that fleas and other biting insects may be concerned in the process appears probable; that poverty, dirt, and lack of ventilation are at least predisposing causes, may be taken as certain; yet notwithstanding the thousands of deaths which have occurred in India every week for a long time past, and the many more thousands of cases which those deaths imply, and notwithstanding the immense field for observation, and if need be for experiment lying open at our hands, we still have to confess that we do not know the mode in which the infection is transferred with sufficient exactitude to be able to make much impression on its dissemination. We manage better even with smallpox and scarlet fever, the microbes of which we hardly yet know by sight. Eleven thousand deaths from plague in India in three weeks, even after all our disinfection. It is worse than disheartening!

